# Influence of Salts on the Photocatalytic Degradation of Formic Acid in Wastewater

**DOI:** 10.3390/ijerph192315736

**Published:** 2022-11-26

**Authors:** Azzah Nazihah Che Abdul Rahim, Shotaro Yamada, Haruki Bonkohara, Sergio Mestre, Tsuyoshi Imai, Yung-Tse Hung, Izumi Kumakiri

**Affiliations:** 1 Graduate School of Sciences and Technology for Innovation, Yamaguchi University, 2-16-1 Tokiwadai, Ube 755-8611, Japan; 2 Department of Oil and Gas Engineering, School of Chemical Engineering, College of Engineering, Universiti Teknologi MARA, Shah Alam 40450, Selangor, Malaysia; 3 Chemical Engineering Department, University Institute of Ceramic Technology, Universitat Jaume I. Avda, Vicent Sos Baynat, 12071 Castellon, Spain; 4 Department of Civil and Environmental Engineering, Cleveland State University, Cleveland, OH 44115, USA

**Keywords:** photocatalysis, Ag/TiO_2_, inorganic salts, immobilization, isoelectric point

## Abstract

Conventional wastewater treatment technologies have difficulties in feasibly removing persistent organics. The photocatalytic oxidation of these contaminants offers an economical and environmentally friendly solution. In this study, TiO_2_ membranes and Ag/TiO_2_ membranes were prepared and used for the decomposition of dissolved formic acid in wastewater. The photochemical deposition of silver on a TiO_2_ membrane improved the decomposition rate. The rate doubled by depositing ca. 2.5 mg of Ag per 1 g of TiO_2_. The influence of salinity on formic acid decomposition was studied. The presence of inorganic salts reduced the treatment performance of the TiO_2_ membranes to half. Ag/TiO_2_ membranes had a larger reduction of ca. 40%. The performance was recovered by washing the membranes with water. The anion adsorption on the membrane surface likely caused the performance reduction.

## 1. Introduction

A large variety of persistent organic molecules are found in the wastewater from municipal and industrial activities of a city. Some of these organics are bio-accumulative and can be toxic, even in low concentrations. However, current technologies have difficulties in treating these dilute components in an economic and environmentally friendly manner. In addition, municipal wastewater contains salinity in the coastal area, which can change the properties of wastewater treatment technology [1,2,3]. For example, salts can hinder the growth of microorganisms and change the performance of activated sludge treatment [1]. Concentrations of 10 to 100 mmol/L NaCl in water changed the adhesion energy of humic substances and affected their removal by adsorption [3]. Coexisting NaCl in water is also reported to slightly reduce catalytic wet-air oxidation activity [2].

Catalytic wet-air oxidation decomposes various types of organics in wastewater that are difficult to degrade by biological treatment [4,5]. Immobilizing catalyst particles on membranes eliminate the process required to separate spent catalysts from the treated water. In addition, the configuration of catalytic membrane contactors facilitates oxygen supply to the reaction field, which enhances the decomposition rate of dissolved organics by catalytic oxidation [6,7]. For example, platinum-based catalytic membranes decomposed dissolved phenols in seawater under mild conditions, showing the potential for membranes to treat wastewater [6]. However, the catalytic performance needs to be improved to reduce the footprint of the membrane unit. As photocatalysts can decompose various types of organics in water [8], immobilizing these photocatalysts on membranes is a possible solution to improve the catalytic membrane performance.

Titanium dioxide (TiO_2_) is a commonly used photocatalyst as it is robust, nontoxic, and cost-effective. However, TiO_2_ requires UV light irradiation to activate due to the wide band gap energy of 3.2 and 3.0 eV for the anatase and rutile phases, respectively [9,10]. Large efforts have been made to narrow the band gap by modifying TiO_2_ with dye, noble metals, such as Au, Ag, and Pt, transition metals, such as Fe and Cu, and other materials [10]. Successful modifications made the TiO_2_-based catalysts visible-light active and the high performance of decomposing dissolved organics in water is reported [11,12].

Many studies evaluating photocatalytic activity use solutions prepared by dissolving a controlled amount of target organic compounds into distilled water. While salinity can exist in wastewater, its influence on photocatalytic activity is not fully understood. For example, Chen et al. reported a negative influence of anions, such as chloride, on the photocatalytic oxidation of dichloroethane, as anions inhibit the adsorption of dichloroethane to a TiO_2_ surface [13]. On the contrary, Makita et al. reported a positive influence of cations on the decomposition rate of dye [14].

In this study, the influence of water salinity was examined using TiO_2_ and Ag/TiO_2_ membranes. Formic acid was used as a model organic compound in water as its degradation process is simple and easy to follow [15,16]. Salinity in municipal wastewater can increase in the coastal area due to the infiltration of seawater. To simulate the seawater contamination, sodium chloride (NaCl) and magnesium sulfate (MgSO_4_) were used as salts. Magnesium chloride (MgCl_2_) and potassium sulfate (K_2_SO_4_) were also used for comparison.

## 2. Materials and Methods

Porous flat discs were prepared using kaolin (ER, Caobar S.A., Guadalajara, Spain), alumina (AR12B5, Aluminium Pechiney, Salindres, France), potato starch (Sigma Aldrich Inc., St. Louis, MO, USA) and polyvinyl alcohol (PVA, Mowiol 4-88, Sigma Aldrich Inc., USA) as a ligand. After sintering the supports at 1673 K for four hours, XRD analysis identified mullite, corundum, and cristobalite (Figure S1). TiO_2_ particles (P25, Evonik Industries) were applied to the surface of the disc supports, having a 47 mm diameter. A detailed procedure will be found elsewhere [15]. Then, silver was photochemically applied to some of the prepared TiO_2_ membranes using a silver acetate solution of 0.01–1 mmol/L. The concentration of silver in the immersed solution was measured by inductively coupled plasma (ICP, SII Nano Technology Co. Ltd., Tokyo, Japan) before and after applying UV light to the membrane. The amount of silver deposited on the TiO_2_ membranes was calculated from the concentration decrease in silver ions in the solution and the weight of the solution used. Three pieces of black lamps (Toshiba, maximum light emission at 352 nm) were used as the UV source and the light intensity was adjusted to 3.3 mW/cm^2^ by changing the distance between the membrane and the lamps. The light strength was measured by a photometer (C10427H102428, Hamamatsu Photonics, Tsukuba, Japan). Prepared TiO_2_ and Ag/TiO_2_ membranes were analyzed by scanning electron microscope (FE-SEM, JSM-633F, JSM-7600FG, JEOL Ltd., Tokyo, Japan) and X-ray diffraction (XRD, XRD-6100, SHIMADZU Co., Kyoto, Japan) with Cu-Kα radiation.

**Figure 1:**
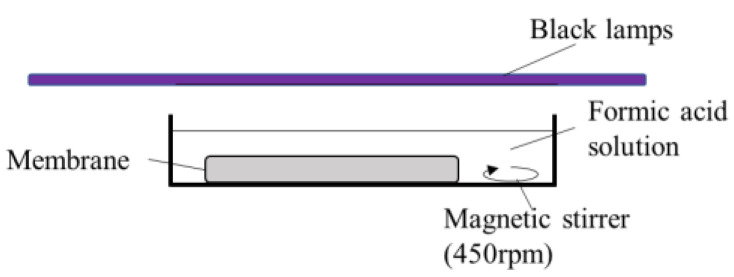
The experimental setup for photocatalytic formic acid decomposition.

The photocatalytic activity of the obtained TiO_2_ and Ag/TiO_2_ membranes was evaluated by the decomposition of formic acid (Fujifilm Wako Pure Chemical Corporation, Osaka, Japan) in water. Formic acid was used in this study. The flat disc-shaped membrane was soaked in formic acid solution and kept under dark conditions for 20 min before applying UV light with a light intensity of 3.3 mW/cm^2^ at room temperature. This light intensity was used to simulate the UVA part of the sunlight [17]. The same black lamps being used in the silver deposition were used as the UV light source in the photocatalytic activity tests. Figure 1 illustrates the experimental setup. Humidity was not controlled nor measured. The starting concentration of the formic acid solution was adjusted to 200 mg/L, a concentration similar to other studies [15]. The concentration of the formic acid solutions was measured using a UV–Vis spectrophotometer UV-1800 (Shimadzu, Columbia, MD, USA) at a 205.6 nm wavelength. Calibration curves to determine the formic acid concentration were prepared for each salt concentration.

Inorganic salts, NaCl, MgSO_4_, MgCl_2,_ and K_2_SO_4_, were purchased from Fujifilm Wako Pure Chemical Corporation, Japan, and dissolved into the formic acid solution at the concentration of 0.6, 6, 60 mmol/L for NaCl, MgSO_4_ and K_2_SO_4_, and 0.3, 3, 30 mmol/L for MgCl_2_. The maximum concentration of NaCl studied was about 1/10 of that in seawater. After each test, the membranes were washed with water.

## 3. Results and Discussion

### 3.1. Influence of Silver Deposition

**Figure 2:**
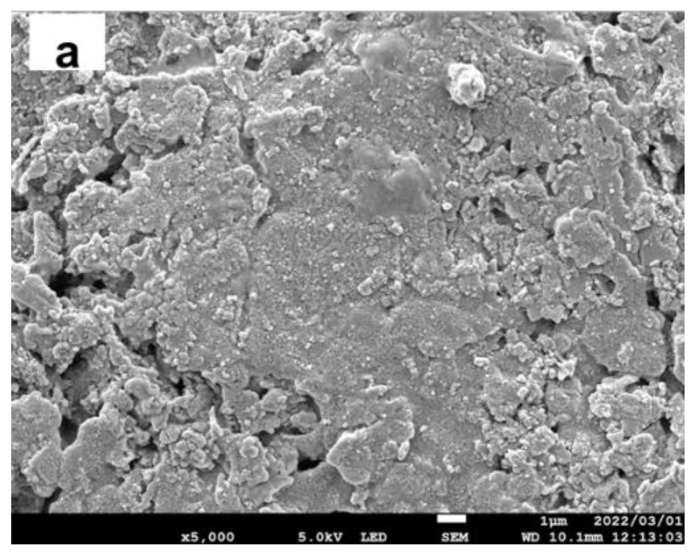

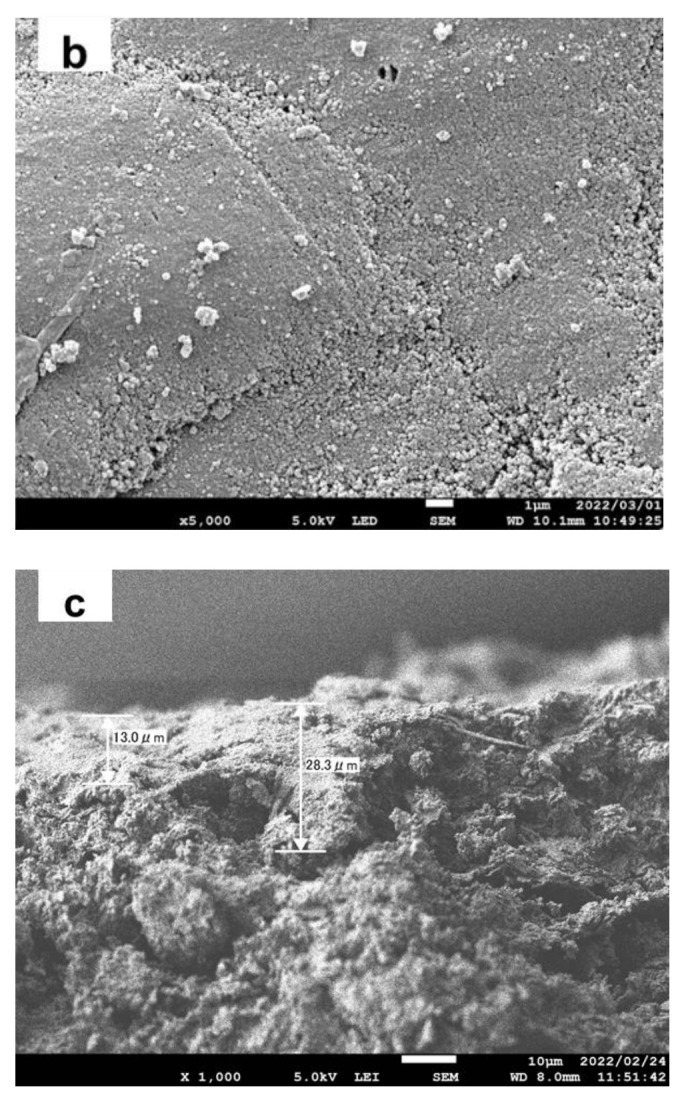
Membrane morphologies (**a**) surface view of the porous ceramic disc, (**b**) surface view of the TiO_2_ membrane, (**c**) cross-sectional view of the TiO_2_ membrane.

Figure 2 shows the membrane morphology. The surface of the porous ceramic disc (Figure 2a) was completely covered with TiO_2_ particles (Figure 2b). The thickness of the TiO_2_ layer was about 10–30 μm (Figure 2c). The variation is due to the large pores in the ceramic disc, which were plugged by the TiO_2_ particles. Element mapping of the cross-sectional view of the TiO_2_ membrane is shown in Figure S2. Al and Si mapping images show a void in the flat support. The Ti mapping showed that such voids can be filled with TiO_2_ particles applied to the surface. As a result, the surface of the TiO_2_ membrane became smoother compared to the support surface (Figure 2a,b). The surface morphology of the AgTiO_2_ membrane was similar to that of the TiO_2_ membrane. No peaks relating to Ag or AgO_x_ were found in the XRD pattern, probably due to its small amount and size [18] (Figure S1). X-ray photoelectron spectroscopy (XPS) analyses showed that silver was deposited as Ag^0^, Ag^+^, and Ag^2+^ (Figure S3).

**Figure 3:**
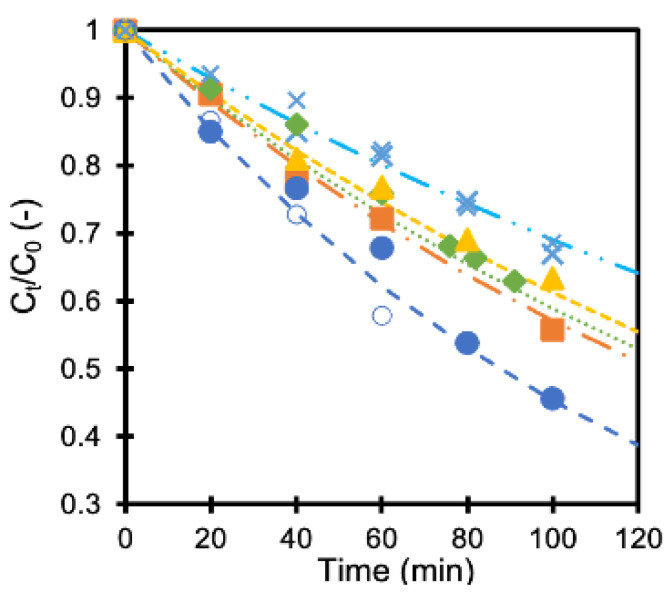
Normalized formic acid concentration as a function of time (x: TiO_2_ membranes, x: TiO_2_ membranes 2nd run, ■ AgT1 membrane, ● AgT2 membrane, ○ AgT2 membrane 2nd run, ◆ AgT3 membrane, ▲ AgT4 membrane).

The concentration of formic acid did not change in the dark, showing little influence on the adsorption of formic acid on the membranes. On the contrary, the concentration decreased when UV light was irradiated, as shown in Figure 3. The concentration at time t (C_t_) was normalized with the initial concentration (C_0_) in the figure. The results obtained with a TiO_2_ membrane and four pieces of Ag/TiO_2_ membrane having different deposition amounts of silver are shown in the same figure. The formic acid concentration became about 75% and 50% compared to the initial concentration after 80 min with TiO_2_ membrane and Ag/TiO_2_ (AgT2) membrane, respectively. Silver deposition improved the photocatalytic activity of the membrane. The membranes showed reproducible performance, as indicated with smaller keys.

The concentration change was fitted (lines in Figure 3) with the first-order equation (Equation (1)) as reported earlier [16].

C_t_/C_0_ = e^−kt^ (1)

where k is a rate constant, and C_0,t_ are the formic acid concentrations at the start and at a time, t, respectively. The calculated rate constant values are summarized in Table 1. Applying small amounts of silver to the TiO_2_ membranes improved the formic acid decomposition rate as reported earlier, for it reduces the recombination of an electron-hole pair that facilitates the formation of oxidants [16,19]. However, an optimum amount of silver maximizes the rate constant. An excessive amount of silver may act as the center for electron-hole recombination, which is considered to be one of the reasons for its negative influence [20].

### 3.2. Effect of Salinity on the Photocatalytic Performance

**Figure 4:**
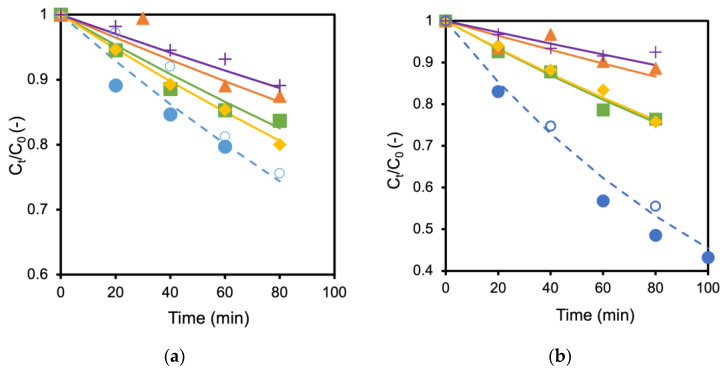
Influence of different salts toward formic acid decomposition; (**a**) TiO_2_ membrane, (**b**) Ag/TiO_2_ membrane; ● No salt, ■ with 60 mmol/L NaCl, ◆ with 30 mmol/L MgCl_2_, ▲ with 60 mmol/L MgSO_4_ and + with 60 mmol/L K_2_SO_4_, ○ membrane performance after washing (no salt).

The TiO_2_ membrane and the Ag/TiO_2_ (AgT2) membrane showing the fastest decomposition rate were used to examine the influence of coexisting salts. Figure 4a,b show the influence of adding different types of salts to the formic acid solution in which TiO_2_ and Ag/TiO_2_ membranes were immersed, respectively. The decomposition of formic acid became slower when the salts were added to the solutions. For example, the decomposition rate became about half when 60 mmol/L NaCl coexisted in the formic acid solution (Figure 4a). Adding 60 mmol/L NaCl and 30 mmol/L MgCl_2_ to the formic acid solutions caused almost the same reduction in the decomposition rate for both TiO_2_ and Ag/TiO_2_ membranes. Adding MgSO_4_ and K_2_SO_4_ further hindered the formic acid decomposition. Interestingly, the performances were recovered by washing the membranes with water (open keys in the figure as control). These results suggest that anions, SO_4_^2−^ and Cl^−^, inhibit formic acid decomposition. A similar reduction by the inorganic salts was observed with TiO_2_ and Ag/TiO_2_ membranes prepared on alumina supports. Consequently, the support materials seem to have a negligible influence on the effect of salts.

**Figure 5:**
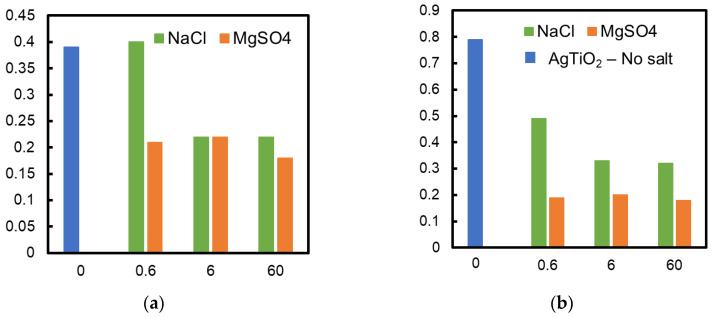
Influence of salt concentration on the rate constant (**a**) TiO_2_ and (**b**) Ag/TiO_2_ membrane; results obtained without any salt addition and addition of NaCl and MgSO_4_ at different concentrations are shown in the figure.

Figure 5 shows the influence of NaCl and MgSO_4_ concentrations on the rate constant of formic acid decomposition. Adding MgSO_4_ to the formic acid solution reduced the photocatalytic activity of both TiO_2_ and Ag/TiO_2_ membranes. The MgSO_4_ concentration did not affect the decomposition rate. Both TiO_2_ and Ag/TiO_2_ membranes showed almost the same rate constant when MgSO_4_ was added, showing no relevant enhancement of depositing silver to the TiO_2_ membrane. A NaCl concentration greater than 6 mmol/L in the solution hindered the decomposition of formic acid by the TiO_2_ membrane to the same degree as MgSO_4_. On the contrary, NaCl addition showed less influence on the Ag/TiO_2_ membrane performance. The decomposition rate with the latter was higher compared to that with the TiO_2_ membrane when membranes were applied in solutions of the same NaCl concentration.

Since chloride enhances silver dissolution [21], this process under experimental conditions was checked. Membranes were prepared under the same conditions as for the AgT2 membrane in Table 1. The membranes were soaked in a 40 g solution for one hour and the concentration of silver was measured by ICP. Silver dissolution was enhanced about 8 times in the NaCl-containing solution under dark conditions. The dissolution was almost negligible under UV light (Table 2).

**Figure 6:**
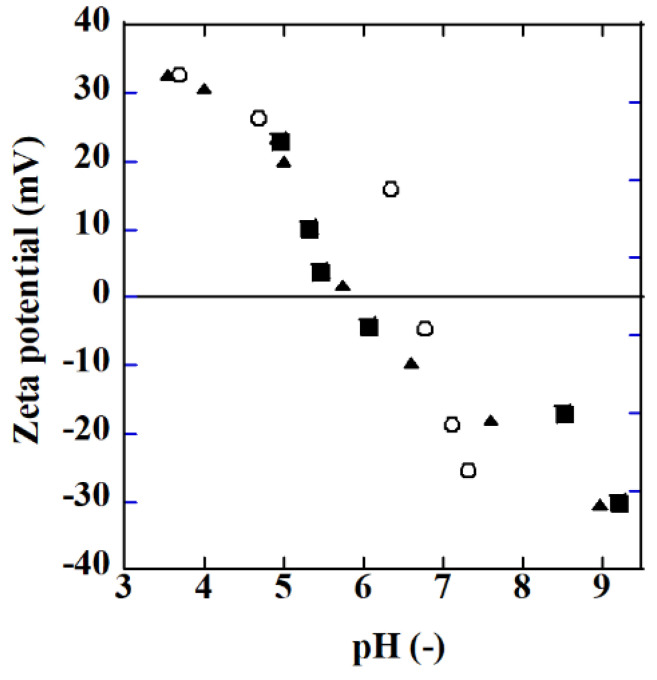
Zeta potential as a function of pH (○: TiO_2_ (P25), ■: Ag/TiO_2_ (0.89 mg Ag/g TiO_2_), ▲: Ag/TiO_2_ (8.9 mg Ag/g TiO_2_).

Figure 6 shows the zeta potential of TiO_2_ (P25) particles and Ag/TiO_2_ particles measured using a Zetasizer Ultra (Malvern Instruments Ltd., Malvern, UK). HNO_3_ and NaOH were used to adjust the pH of the solution. The Ag/TiO_2_ particles were prepared in the same manner as for membrane preparation. The amount of silver on TiO_2_ particles, 0.89 and 8.9 mg Ag/g TiO_2_, were comparable to the membranes. For example, the AgT2 membrane in Table 2 had a silver amount of ca. 2.5 mg Ag/g TiO_2_.

The isoelectric point (IEP) of TiO_2_ was about 6.5, which is a value reported similarly in [22]. The IEP value became smaller with silver deposition on TiO_2_. The difference in the silver amount did not affect the IEP. The pH of the formic acid solution used to evaluate the photocatalytic property of the membranes changed from ca. 3.1 to 3.3 during the decomposition of the acid. Accordingly, the surface of both the TiO_2_ and Ag/TiO_2_ membranes was positively charged when immersed in the formic acid solution. The SO_4_^2−^ and Cl^−^ anions coexisting in the solution can adsorb on the membrane surface and may hinder the photocatalytic reactions by hindering the adsorption of hydroxide to the surface which reduces the formation of oxidants, for example.

## 4. Conclusions

Ag/TiO_2_ membranes were prepared by the photochemical deposition of silver on TiO_2_ membranes. A small amount of silver deposition improved the photocatalytic decomposition rate of formic acid dissolved in wastewater. The excessive addition of silver reduced the decomposition rate. The largest enhancement was with 2.5 mg Ag/g TiO_2_. Salinity in water reduced the decomposition property of both the TiO_2_ and Ag/TiO_2_ membranes, but the membrane performance recovered by washing the membrane with water. The addition of NaCl influenced the Ag/TiO_2_ membranes less than the TiO_2_ membranes. The reduction in oxidation performance can be attributed to the anion adsorption on the membrane surface, which is positively charged in a formic acid solution. Ag deposition on TiO_2_ shifts the IEP value to lower pH, which may reduce the anion influence at around a pH of 6. The zeta potential cannot explain the different influences of SO_4_^2−^ and Cl^−^ on the Ag/TiO_2_ and TiO_2_ membranes and further study is required to understand this mechanism. Even though the decomposition rate became about half when using the coexisting salts, photocatalytic membranes decomposed dissolved formic acid. The results suggest a wider application potential of photocatalytic membranes.

## Figures and Tables

**Table 1:** Influence of silver on the rate constant values obtained in formic acid decomposition.

Membrane No.	Mass of Silver (mg)	k × 10^2^ (min^−1^)	Coefficient of Determination (R^2^)
TiO_2_	0	0.39	0.942
AgT1	0.03	0.57	0.962
AgT2	0.15	0.78	0.976
AgT3	0.37	0.50	0.963
AgT4	3.1	0.48	0.975

**Table 2:** Silver dissolution from Ag/TiO_2_ membrane in different solutions.

Solutions	Silver Concentration (mg/L)	
	Dark	UV Irradiation
Formic acid	0.011	0.0038
Formic acid + NaCl	0.088	0.0037
Formic acid + MgSO_4_	0.015	0.0034

## Data Availability

Not applicable.

## Supplementary Materials

The following supporting information can be downloaded at: https://www.mdpi.com/article/10.3390/ijerph192315736/s1. Figure S1: XRD patterns (a) porous ceramic disk, (b) TiO_2_ membrane and (c) AgTiO_2_ membrane; Figure S2: SEM-EDS images of AgTiO_2_ membrane (cross sectional view) (a) cross sectional image, (b) Al mapping, (c) Si mapping, (d) Ti mapping; Figure S3: XPS of TiO_2_ membrane and AgTiO_2_ membrane (a) wide scan spectra, (b) O_1s_ spectra, (c) Ti_2p_ spectra, and (d) Ag_3d_ spectrum of AgTiO_2_ membrane with fitting curves.
