# Supplementary material for: Influence of Salts on the Photocatalytic Degradation of Formic Acid in Wastewater
Source: Int J Environ Res Public Health. 2022 Nov 26;19(23):15736. doi: 10.3390/ijerph192315736 (PMC9737836; doi:10.3390/ijerph192315736)
Supplement: Supplementary file 1 [file ijerph-19-15736-s001.zip › ijerph-2028087-supplementary.pdf]

International Journal of
Environmental Research and Public Health

Supplementary Materials 1

XRD patterns of the porous ceramic disk, the TiO_2 membrane and the $AgTiO_2$ membrane. The porous supports were made of mullite, corundum, and cristobalite. The peaks related to the supports became smaller in TiO_2 and $AgTiO_2$ membranes due to the TiO_2 layer applied at the surface of the support. The XRD patterns obtained with TiO_2 and $AgTiO_2$ membranes were the same.

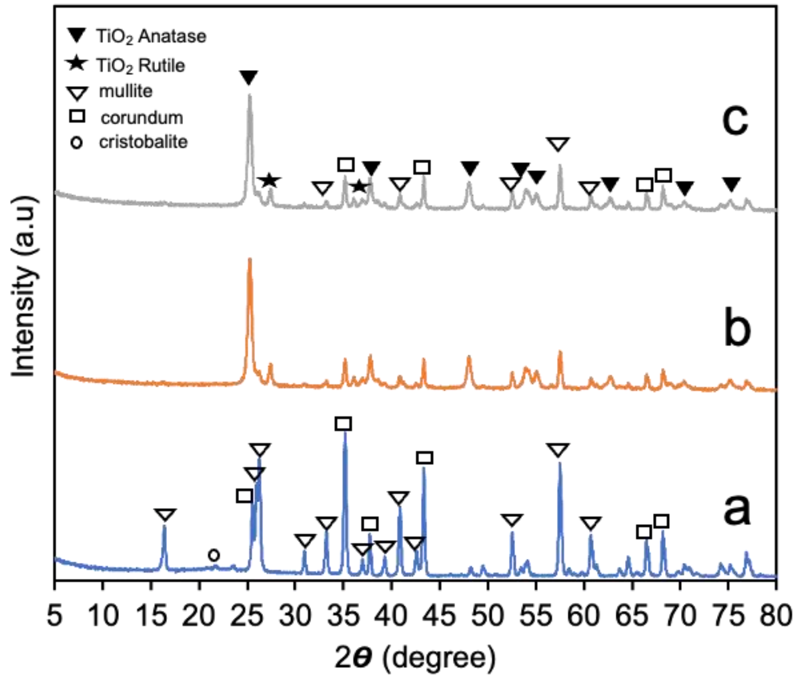

Figure S1 XRD patterns a) porous ceramic disk, b) TiO_2 membrane and c) $AgTiO_2$ membrane

Supplementary Materials 2

The aluminum and silicon found in the SEM-EDS images of a $AgTiO_2$ membrane correspond to the support material. Titanium is mainly distributed at the top surface of the membrane.

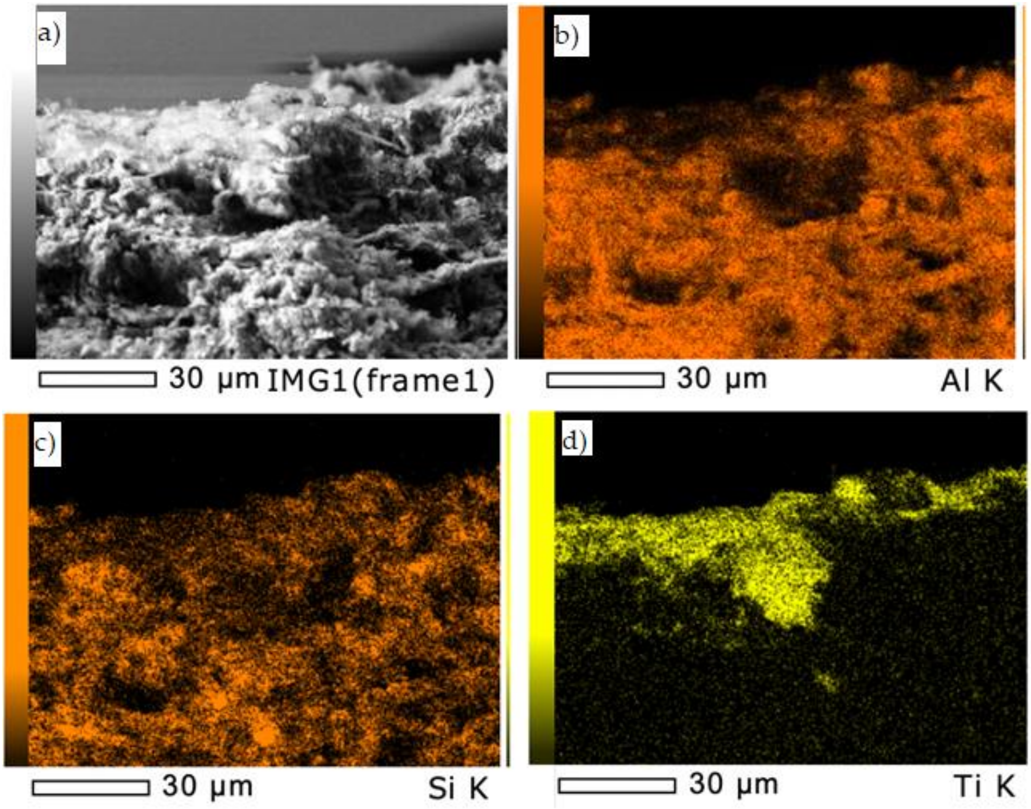

Figure S2 SEM-EDS images of $AgTiO_2$ membrane (cross sectional view) (a) cross sectional image, b) Al mapping, c) Si mapping, d) Ti mapping)

Supplementary Materials 3

1. The state of silver photochemically deposited to TiO_2 membranes was analyzed by X-Ray Photoelectron Spectroscopy (XPS, Thermoscientific K-Alpha X-ray, Japan) using AlKα radiation of 1486.6 eV. The C_{1s} binding energy peak at 284.8 eV was used for the charge correction. Thermo Scientific Avantage software was used for the fitting.

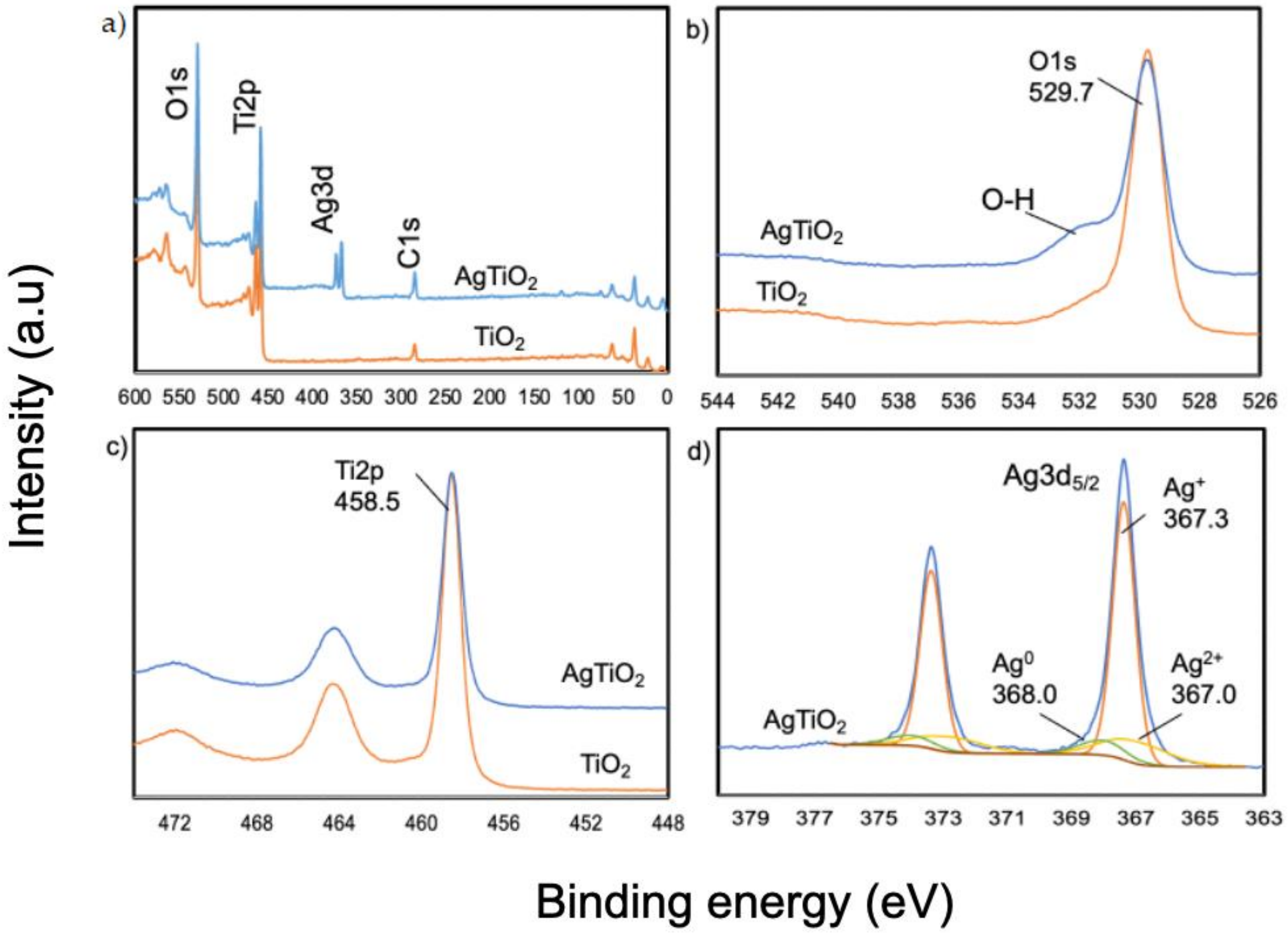

FigureS3 XPS of TiO_2 membrane and $AgTiO_2$ membrane a) wide scan spectra, b) O_{1s} spectra, c) Ti_{2p} spectra, and d) Ag_{3d} spectrum of $AgTiO_2$ membrane with fitting curves
